# Oh nuts, they’ve got a pelvic kidney – a tricky testicular vein embolisation

**DOI:** 10.1259/bjrcr.20220130

**Published:** 2023-04-19

**Authors:** Sajal Patel, Irfan Ahmed, Benedict Thomson

**Affiliations:** 1 1 Interventional Radiology Department, Guy’s and St Thomas’ NHS Foundation Trust, St Thomas' Hospital, London, United Kingdom

## Abstract

Testicular vein embolisation for varicocele is a common interventional procedure performed in predominantly young, healthy males. Cross-sectional imaging is rarely performed for treatment planning and is often not available. In this case report, we describe a case of testicular vein embolisation in an ipsilateral pelvic kidney where cross-sectional imaging aided treatment planning resulting in successful embolisation.

## Summary

A varicocele is an abnormal dilatation of the pampiniform plexus secondary to testicular vein incompetence. It may cause subfertility and testicular pain or discomfort. The left testicular vein usually drains into the left renal vein and it is the unfavourable angle of this junction which often leads to a left-sided varicocele due to increased hydrostatic pressure.^1^ Multiple anatomical variants of the left testicular vein^2^ have been described in the context of testicular vein embolisation and variants of the inferior vena cava (IVC) and its tributaries^3^ have also been described. However, there are no reports of testicular vein embolisation in congenitally anomalous kidneys. We present a case of percutaneous varicocele embolisation in a patient with a painful varicocele and an ipsilateral pelvic kidney.

## Clinical presentation

A 77-year-old male was seen by Urology with worsening left testicular pain. He underwent a left inguinal hernia repair 2 years previously and since then had noticed increasing pain. The pain was described as dull and squeezing with no relieving factors. On examination, there was a Grade 2 left-sided varicocele. An ultrasound (US) was performed which reported a mild varicocele with a maximal diameter of 2.9 mm. Open surgical and endovascular options were discussed with the patient, he opted for endovascular therapy and was referred to Interventional Radiology for a left testicular vein embolisation.

During pre-assessment work-up, historical imaging from an external hospital was reviewed and it was noted that the patient had a left-sided pelvic kidney. On the morning of the procedure, the patient underwent a portovenous phase CT scan in order to map the anatomy and determine the origin of the left renal vein and potentially the left testicular vein.

## Imaging findings

**Figure 1.**
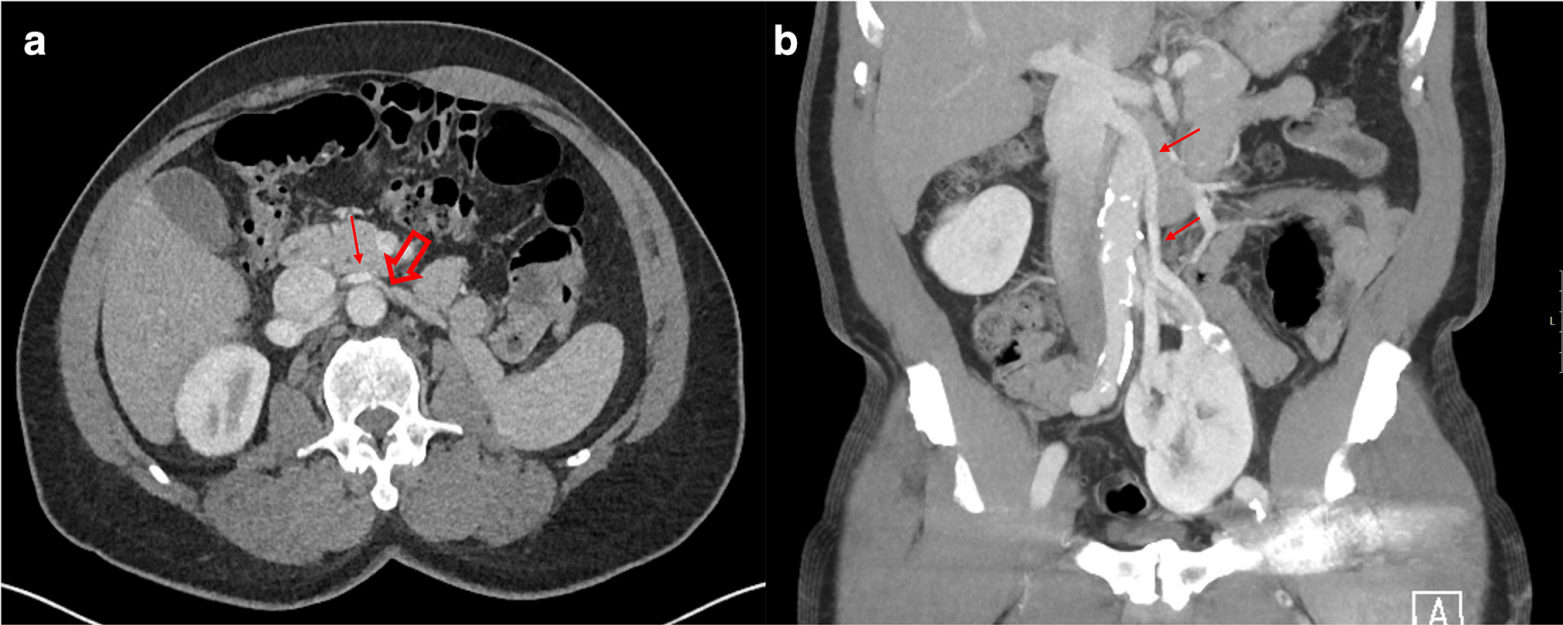
(**a**) Axial image of portovenous CT at the level of L2. Solid red arrow demonstrating the left lower pole renal vein branch passing anterior to the aorta. Open red arrow demonstrating a small branch arising from the left lower pole renal vein thought to be a candidate for the left testicular vein. (**b**) Coronal reformat demonstrating the course of the lower pole renal vein.

A CT scan performed with a 70 second delay demonstrated the left pelvic kidney and the venous drainage. The lower pole drained through two branches which join just above the level of the renal hilum to take a course anterior to the aorta and drain into the IVC at the level L1/L2 (Figure 1). The upper pole is drained by a vein that takes a retro-aortic course and drains into the IVC at the level of L3/4.

**Figure 2.**
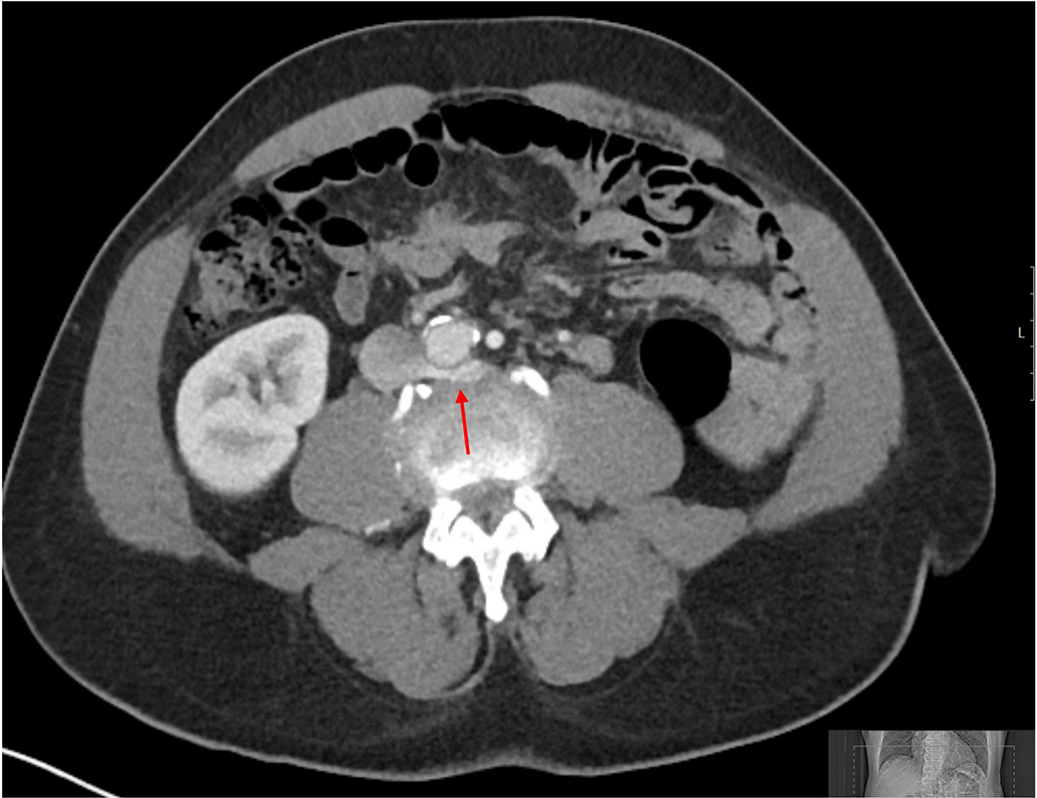
Axial image of portovenous CT at the level of L3/4. Red arrow demonstrating the left upper pole renal vein branch taking a retroaortic course with mild compression.

**Figure 3.**
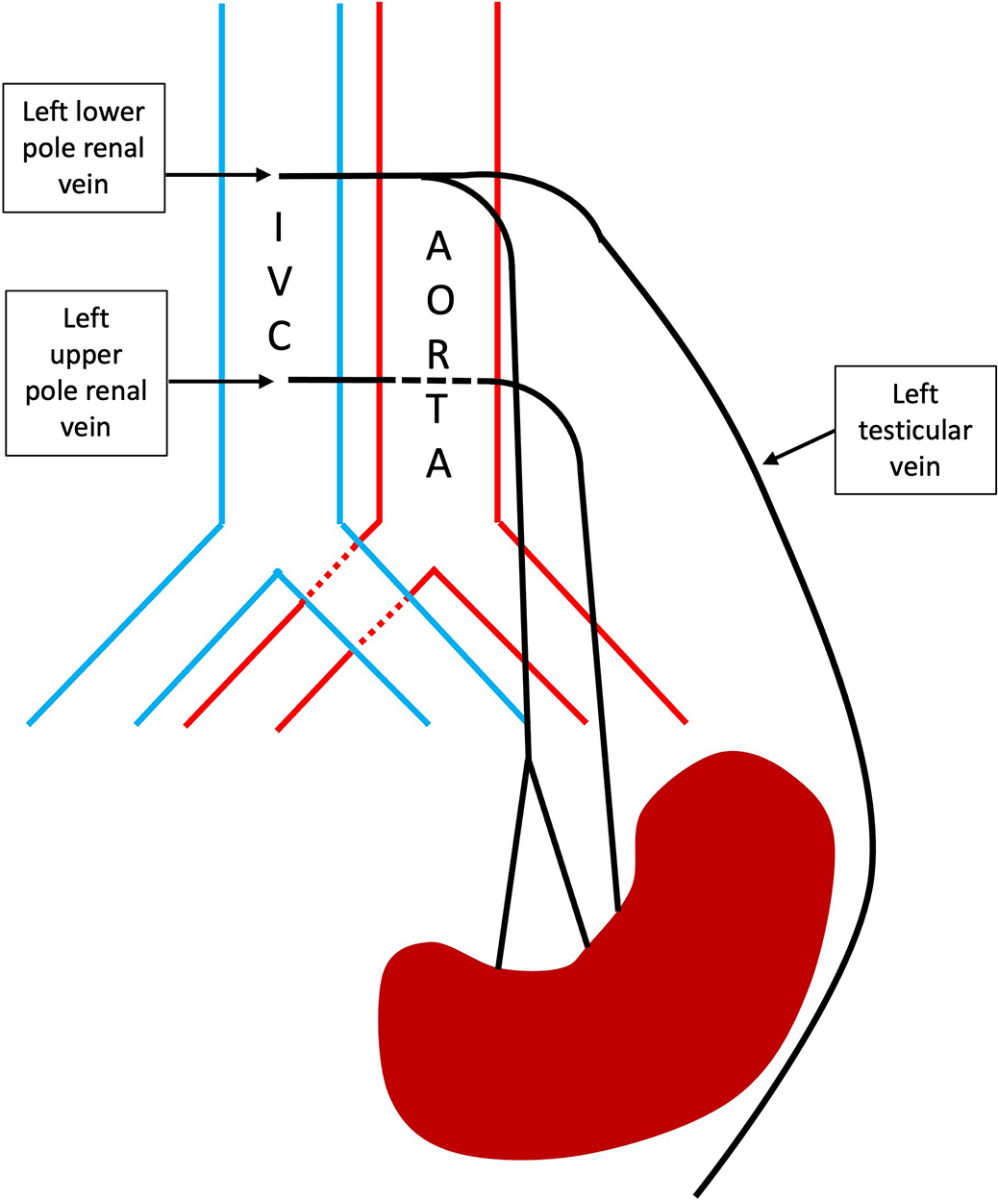
A schematic representation of the variant anatomy.

Both renal veins were interrogated to determine their course from the IVC and any branches which may represent the left testicular vein. The upper pole vein takes a horizontal course posterior to the aorta before taking a shallower angle to pass inferiorly towards the kidney. There was some compression of the retro-aortic portion of the vessel (Figure 2). The lower pole vein demonstrated a small branch which arose at the midline at the level of L2 and passed around the aorta towards the left flank, this vessel was felt to represent the best candidate for the left testicular vein (Figure 1). The vessel then coursed inferiorly towards the left lower quadrant for a short distance beyond which it could not be traced. Figure 3 demonstrates a schematic depiction of the variant anatomy. No further branches were identified and there were no other potential candidates for the left testicular vein.

## Treatment

The patient was informed of the CT findings and advised that the abnormal anatomy will increase the risk of procedural failure. A plan was made that the lower pole renal vein, seen to arise at the midline, would be cannulated first. Should the cannulation of this vein fail or should there be no reflux into the testicular vein, the upper pole renal vein would be targeted.

**Figure 4.**
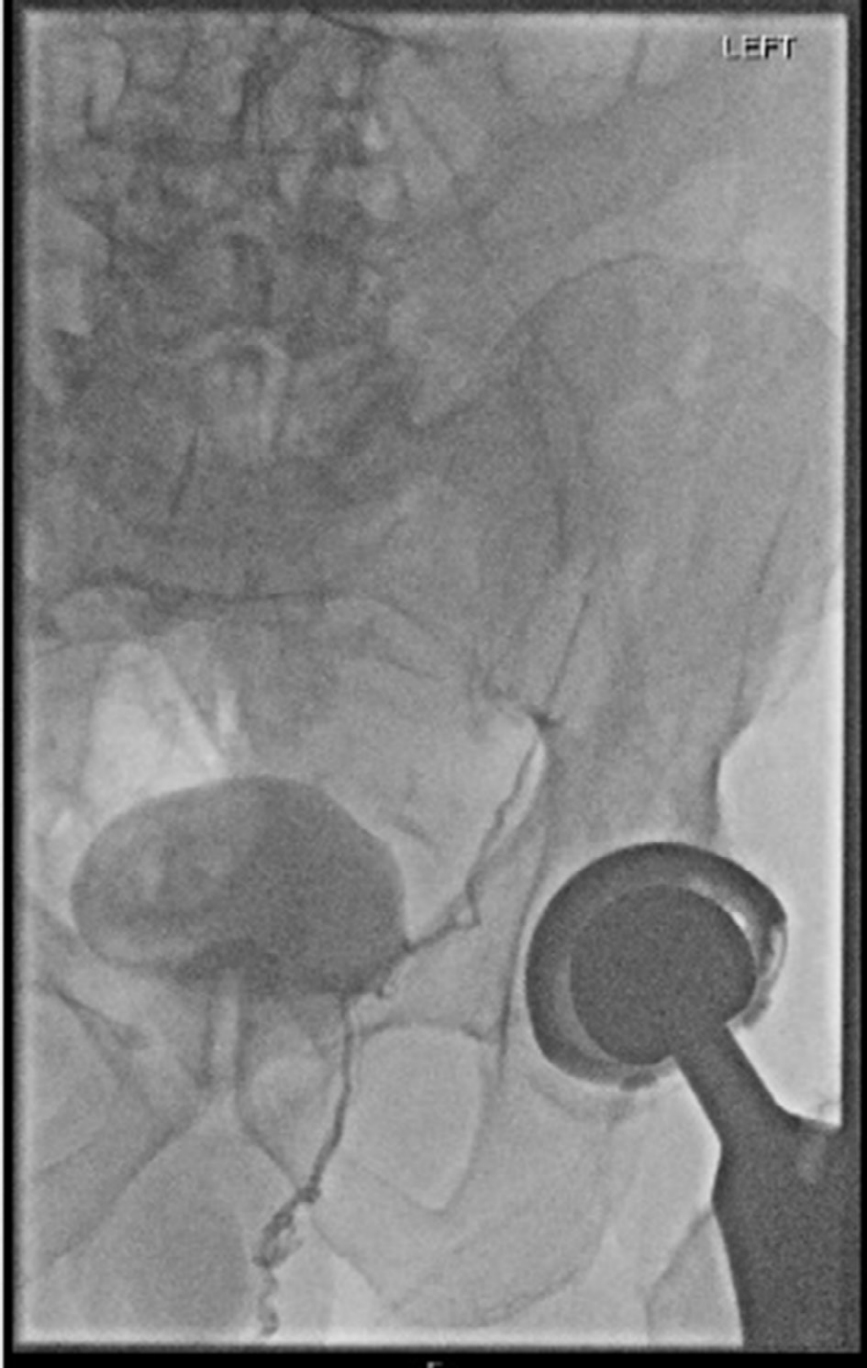
Fluoroscopic image of contrast filling a left testicular vein.

Due to the downward angle of the left lower pole renal vein, right internal jugular venous access was secured and a 5Fr sheath inserted. A 5Fr multipurpose catheter was used to cannulate the left lower pole renal vein. The tip of the catheter was directed posterolaterally at the level of L2 after correlating with the CT imaging. Contrast was injected with small manipulations of the tip of the catheter until contrast was seen to fill a small calibre vessel tracking inferiorly and refluxing below the inguinal ligament (Figure 4). A 2.7Fr microcatheter (Progreat, Terumo Corporation) was used to advance further into the vein to avoid vessel spasm or injury. Further contrast injection demonstrated a single left testicular vein.

**Figure 5.**
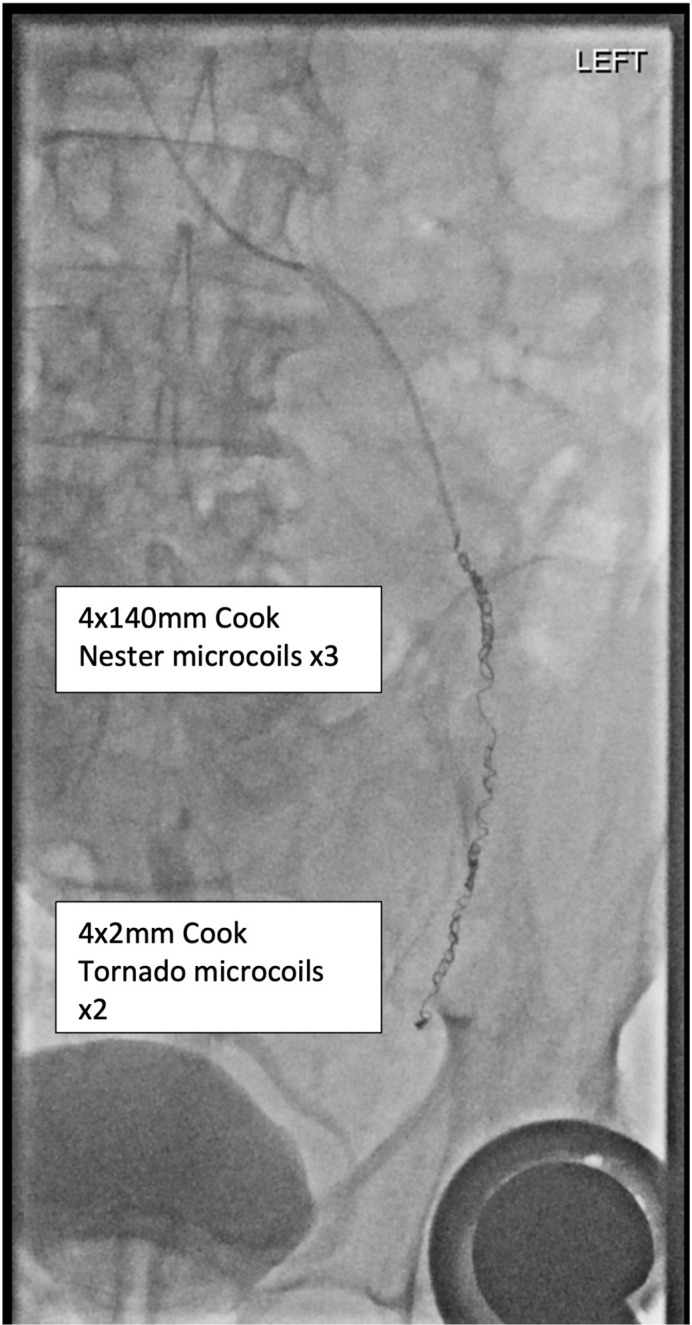
Final venographic image following administration of multiple coils with no reflux beyond them towards the pelvis.

Two 4 × 2 mm microcoils (Tornado, Cook Medical) were deployed just inferior to the level of the sacroiliac joint. Contrast injection at this point demonstrated persistent reflux below the level of the inguinal ligament and a branch arising from the level of the mid-sacroiliac joint, passing laterally. The collateral was not seen to drain into the main left renal vein and may, therefore, represent a Class four configuration draining into renal capsular veins.^2^ Following further deployment of three 4 mm x 14 cm microcoils (Micro Nester, Cook Medial), the collateral branch no longer filled and there was no reflux beyond the coils (Figure 5).

Given that a radiologic endpoint of embolisation was reached the decision was made to end the procedure there without attempting to cannulate the posterior renal vein due to the perceived potential difficulties. If symptoms persisted, a repeat procedure could be attempted with an effort to interrogate the retro-aortic renal vein.

## Outcome and Follow-Up

There were no access or embolisation-related complications in the immediate post-operative period. Furthermore, the patient reported a significant improvement in his symptoms with a substantial reduction in testicular pain, reduced prominence of the testicular veins and reduced scrotal swelling two months following the procedure.

## Discussion

Varicocele is considered by some to be a leading reversible cause of male infertility.^4^ Percutaneous testicular vein embolisation offers a minimally invasive, non-surgical therapeutic option for the treatment of varicoceles and has also been shown to be efficacious in the treatment of painful varicoceles.^5^ Pertinent complications of the procedure include procedural failure, recurrence of varicocele and non-target embolisation. Retrograde venography performed at the time of percutaneous embolization can determine the classification of a varicocele and guide therapeutic decisions. Anterograde venography, while feasible, is not routinely performed in the traditional surgical approach.^2^ The presence of untreated collaterals can result in surgical and percutaneous failure.^4^

A variety of different embolic agents are available including coils, sclerosants and other liquid embolics. These have all been shown to be safe and effective in the treatment of varicoceles.^6^ Depending on the complexity of the varicocele and the number of collateral feeding vessels one may decide to use a liquid embolic. Or in more simple cases such as this where there were no collaterals, coils alone are sufficient.

A pelvic kidney results from an error in embryological development during the ascent of the kidney and is commonly associated with variant vasculature.^7^ Identification of anatomical variants is key to any interventional or surgical procedure. At our centre, standard pre-operative imaging prior to testicular vein embolisation is a testicular US with the addition of a renal US if there is reason to suspect a secondary cause of varicocele. This case demonstrates the need for the addition of routine renal US to confirm the position of the left kidney. The left testicular vein usually drains into the left renal vein^8^ and can be cannulated from right common femoral access however right internal jugular vein access can be helpful for cannulation of the right testicular vein^9^ or in our case for variant anatomy. Despite the extra radiation exposure, cross-sectional imaging can reduce procedural time and thereby radiation dose by indicating potential landmarks in the case of variant anatomy and reduce the time it takes to cannulate target vessels.

Left renal vein entrapment has been associated with varicocele recurrence following surgical repair;^10^ however, there is no published data regarding the outcomes of percutaneous embolisation in the same clinical setting. The mechanism for recurrence following surgical repair, increased left renal vein pressures causing increased pressures within the left testicular vein, is also feasible in patients treated percutaneously especially if small collaterals or duplicated veins have not been identified and treated in the initial procedure.

Due to the variant anatomy in this case, there was a perceived high risk of failure prior to commencing the procedure. The patient was counselled appropriately at the time of the procedure and measures to ensure comfort were undertaken prior starting a potentially prolonged case. The cannulation of the left testicular vein was, however, straightforward and the patient tolerated the procedure under local anaesthetic very well. Although the vein itself was not significantly enlarged, by virtue of it causing a symptomatic Grade two varicocele, it was amenable to cannulation.

## Learning points

1. Variant anatomy can pose difficulties when performing image-guided procedures reliant on accepted anatomical configurations.
2. Performing cross-sectional imaging to plan the approach can save valuable time and radiation on the fluoroscopy table.
3. Being aware of anatomical variants can not only help plan the procedure but can also help guide when to stop in the case of chasing the left retro-aortic renal vein branch.
4. Despite the perceived difficulties of the case, a traditional approach may still succeed and is worth trying in the first instance.
